# Can Emotion Regulation Affect Aggressive Responses? A Study on the Ukrainian–Russian Conflict in a Non-Directly Exposed Sample

**DOI:** 10.3390/ijerph19106189

**Published:** 2022-05-19

**Authors:** Clarissa Cricenti, Emanuela Mari, Benedetta Barchielli, Alessandro Quaglieri, Jessica Burrai, Alessandra Pizzo, Ivan D’Alessio, Anna Maria Giannini, Stefano Ferracuti, Giulia Lausi

**Affiliations:** 1 Department of Psychology, Sapienza University of Rome, 00185 Rome, Italy; e.mari@uniroma1.it (E.M.); alessandro.quaglieri@uniroma1.it (A.Q.); jessica.burrai@uniroma1.it (J.B.); alessandra.pizzo@uniroma1.it (A.P.); ivan.dalessio@uniroma1.it (I.D.); annamaria.giannini@uniroma1.it (A.M.G.); giulia.lausi@uniroma1.it (G.L.); 2 Department of Dynamic, Clinical Psychology and Health, Sapienza University of Rome, 00185 Rome, Italy; benedetta.barchielli@uniroma1.it; 3 Department of Human Neuroscience, Sapienza University of Rome, 00185 Rome, Italy; stefano.ferracuti@uniroma1.it

**Keywords:** emotions, emotional intelligence, intergroup conflict, moderated mediation, outgroup, ingroup

## Abstract

On 24 February, Russian President Vladimir Putin gave the order to invade neighbouring Ukraine; a typical trend during the war is considering events in a one-sided way, emphasising the exclusive contribution of one opponent over the other for the outbreak of war. War may trigger the experience of emotions, such as anger, shame, and disgust. The present study reproduces previous studies on the influence of emotional regulation in support of aggressive reactions (AR) in the Israeli–Palestinian conflict. A questionnaire referring to the Russian–Ukrainian conflict has been implemented and spread in the Italian territory. A multiple moderated mediation model was proposed to evaluate the effect of emotional cognitive reappraisal on the propensity for AR, including conflict-related emotions (anger, shame, disgust) as mediators and political alignment and the appraisal of one’s own emotions subscale of the brief emotional intelligence scale as moderators. The results show that cognitive reappraisal of emotions has a negative effect on AR; moreover, recognising and regulating emotions decreases anger, while taking sides with Ukraine or not siding seems to have an effect on AR depending on the emotion felt (anger or shame). The results are discussed according to the current literature on the topic, highlighting the practical implications and limits of the research.

## 1. Introduction

On 24 February, Russian President Vladimir Putin gave the order to invade neighbouring Ukraine shortly after the official recognition of the Donbas separatist republics located in Ukrainian territory, Donetsk and Lugansk, and the troops that were sent into these territories were regarded as being on a peacekeeping mission. Hostilities have been ongoing for eight years [1], as this country can be recognised as a “buffer area” between Europe and Russia. However, we are also seeing significant effects in Europe, with energy and other prices rising and probably set to continue to do so. In addition, European people are bearing witness to heart-breaking scenes, and European agencies are warning of an impending humanitarian crisis. Regardless of the motivations or groups involved, war presents itself as an intergroup conflict, which has been defined by the American Psychological Association (APA) as a disagreement or confrontation between two or more groups and their members (e.g., between work departments, entire companies, political parties, or nations) and may involve interpersonal discord, psychological tension, or physical violence.

A typical trend during the war is considering events in a one-sided way, emphasising the exclusive contribution of one opponent over the other for the outbreak of war [2]. Whether one reacts to conflict (i.e., adopting aggressive and polarising or reconciliatory attitudes and behaviours) may stem from the emotion experienced at that particular moment [3,4,5,6,7,8,9,10,11,12,13]. Indeed, both individual and intergroup emotions (i.e., expressing emotions towards “outgroup” because of their belonging or identification to a specific group or in response to events that affect the group itself) are defined as brief and transient reactions to an event, which influence thoughts and motivate the enactment of immediate response [14,15,16,17,18].

Indeed, moral emotions affect the relationship between moral norms and moral behaviours, prompting people to act in moral and socially appropriate ways in their social interactions and intimate relationships [19,20]. In the context of intergroup relations and intergroup conflict, anger, shame, and disgust are probably some of the most relevant emotions. Reappraised anger attributes low levels of morality to the outgroup or event, leading to the experience of other emotions, such as hatred and disgust [21]. Moreover, disgust seems to be implicated in dehumanisation [22] and, through a further re-evaluation of the events, a perceived need for the elimination of the outgroup or event [21]. Shame can lead to positive behaviours to repair behaviours experienced as unacceptable, or it may have a destructive impact on social relationships by escalating into forms of violence [23,24,25]. This can occur when shame is perceived as stemming from the actions of others (“others made me feel ashamed”) or from thoughts of powerlessness, when violence is perceived as the only available response (“I had no other choice”) and when the emotional abilities to recognise emotion and inhibit violent impulses are lacking [25,26,27,28]. Given the role of emotions in initiating and maintaining conflict by eliciting aggressive or violent behaviours [7,9,12,21,29,30], the importance of emotional regulation has been investigated in the literature. Emotion regulation has been defined as the process by which individuals shape the intensity and valence of their emotions, when they experience them, and how they are expressed [5]. Specifically, Helperin and colleagues [31] explored the relationship between emotions and cognitive reappraisal (an adaptive emotional regulation strategy) in support of conciliatory and aggressive policies in the context of the Israeli–Palestinian conflict. The findings revealed a positive effect of anger on support for aggressive policies. However, participants who were asked to use a cognitive reappraisal strategy to regulate anger emotion were less supportive of aggressive policies compared to the control group, who was not given any indication of emotional regulation. Hurtado-Parrado and colleagues [32] replicated the same study in the conflict between the Colombian government and the Fuerzas Armadas Revolucionarias de Colombia-Ejército del Pueblo (FARC-EP). Once again, it was found that the participants who used cognitive reappraisal to regulate negative emotions were less favourable towards aggressive policies than the control group. Thus, these studies reveal a mediating effect of emotions in the relationship between cognitive reappraisal and support for aggressive policies in war contexts. Related studies [8,9] have shown that the use of emotional regulation strategies, such as cognitive reappraisal, predicts support for humanitarian aid towards outgroup members even in times of war and, in turn, appears to be related to greater support for conciliatory policies and less support for aggressive policies.

To the best of our knowledge, more focus has been placed on emotional regulation (i.e., cognitive reappraisal and emotional suppression), overlooking the possible role of emotional intelligence (EI) in conflicting attitudes between groups. EI can be described as the “ability to monitor one’s own and others’ feelings and emotions, to discriminate among them and to use this information to guide one’s thinking and actions” [33]. Therefore, EI is a multidimensional construct, including the ability to appraise, express, and regulate one’s own and others’ emotions and to use them in adaptive ways (e.g., problem solving, future planning, focus of attention, motivation). The main function of EI is the ability to adapt to the context, as these skills enable individuals to accurately gauge their own and others’ affective responses to focus cognitive activities and choose socially adaptive behaviours.

Individuals with greater EI seem to prefer collaborative conflict resolution strategies [34,35], and a study by Castillo and colleagues [36] found that a group of students who underwent EI training reported lower levels of physical/verbal aggression, anger, hostility, personal distress, and fantasy than the students in the control group. Moreover, identifying emotions (particularly negative emotions) allows for a decision as to whether to regulate them and is key to facilitating and making this process more effective [37,38,39,40,41]. The study of the relationship between emotional regulation and EI has shown that difficulty in understanding and distinguishing negative emotions would appear to have an effect on affective responses by making them more unpleasant even when “adaptive” emotional regulation strategies are used [42,43,44].

### Aims

The present research aimed to investigate the influence of emotion regulation and EI on aggressive policies towards the current Russian–Ukrainian war and the mediating role of emotions in an Italian sample.

**H1:**  *There is a relationship between cognitive reappraisal and aggressive reactions (AR) mediated by negative emotions*.

**H2:**  *Appraisal of one’s own emotion moderates the relationship between cognitive reappraisal and negative emotions*.

**H3:**  *Political alignment has a moderating effect on the relationship between negative emotion and AR*.

## 2. Materials and Methods

### 2.1. Procedure

Data were collected from 4 March 2022 to 16 March 2022. Participants completed an online survey optimised for use on mobile devices through the Qualtrics platform used to distribute the questionnaire widely throughout Italy. A nonprobabilistic and convenience sampling technique was used to successfully attract as many voluntary participants, who were motivated by interest and curiosity about the research topic, as possible. The questionnaire was distributed through various social networks and official university channels. Each participant gave their informed consent to voluntarily join the research.

All study procedures were carried out in accordance with the Declaration of Helsinki and with the approval of the Institutional Review Board of the Department of Psychology, University of Rome “Sapienza” (protocol number 515/2022).

### 2.2. Materials

For this study, an online questionnaire consisting of several sections was developed. The first part consisted of a brief summary of the demographic data (i.e., age, gender, nationality, educational degree, marital status, work, religious belief) and participants’ perceived level of information about the Russian–Ukrainian conflict (i.e., “how much do you keep informed about the conflict?”) and whether they felt politically aligned. Then, the remainder of the questionnaire included the following measures.

#### 2.2.1. Aggressive Reactions (AR) to Conflict-Related Events

AR consists of 8 items constructed ad hoc by the authors to assess the general support for aggressive policies in the current Russian–Ukrainian conflict. This scale was developed to investigate the attitudes and political orientation of interviewees on events related to the Russian–Ukrainian conflict through items such as “For the war to end, NATO should intervene militarily as soon as possible” or “Ukraine should prohibit Russians in need of medical treatment from entering its territory”. The 8 items on the political orientation of the interviewees were rated on a 5-point Likert scale, from “strongly disagree” (1) to “strongly agree” (5).

#### 2.2.2. Conflict-Related Negative Emotion

This scale was used to measure negative emotions related to the Russian–Ukrainian conflict, built ad hoc by the authors, and evaluates the intensity of anger, disgust, and shame. The 3 items on the emotional activation of the respondents were evaluated on a 5-point Likert scale, from “never” (1) to “always” (5).

#### 2.2.3. Emotional Regulation Questionnaire

The emotion regulation questionnaire (ERQ) [45] is a self-report scale designed to assess emotion regulation strategies, such as cognitive reappraisal and expressive suppression. For this study, the Italian version was used [46]. Participants responded to each of 10 items using a 7-point Likert scale ranging from 1 (strongly disagree) to 7 (strongly agree). Cognitive reappraisal consists of thinking differently about a critical situation to change its meaning to alter one’s emotional experience. Expressive suppression involves decreased regulation of the outward expression of emotions. Six items refer to the subscale for cognitive reassessment: “To feel better (happy/content/relieved/in a good mood), I try to look at things from a different perspective”. Four items refer to the subscale for expressive suppression: “When I’m happy/joyful, I try not to notice it”.

#### 2.2.4. Brief Emotional Intelligence Scale-10

The brief emotional intelligence scale (BEIS-10) [47] explores people’s individual dispositions regarding the exploration of their own and others’ emotions. In this study, the Italian validation of the BEIS-10 was adopted [48]. It consists of a 10-item self-assessment questionnaire that, through 5 subdimensions, investigates the appraisal of one’s own emotions (i.e., ability to recognise one’s emotions and to identify factors that could change them); appraisal of others’ emotions (i.e., ability to interpret emotions in others); regulation of one’s own emotions (i.e., ability to control and regulate one’s emotions); regulation of others’ emotions (i.e., the ability to promote positive feelings in other people); and utilisation of emotions (i.e., people’s ability to use their positive emotions for problem solving). The participants had to express a degree of agreement for each of 10 items according to a 5-point Likert scale ranging from “totally disagree” to “totally agree”.

### 2.3. Participants

A total of 520 participants joined the research; 11 did not give their informed consent and were therefore excluded. A total of 64.8% were women (*n* = 330), and their ages ranged from 18 to 89 years (M = 40.03; SD = 13.83). Overall, 88% of the sample was born in Italy, while the remaining 11.6% was born abroad; 31.8% of the sample was married, 23.8% cohabiting, 20.2% single, 4.1% separated, 1.4% divorced, and 1.4% widowed. Regarding educational degree, 4.1% of the sample had a middle school diploma, 34.2% had a high school diploma, 18.0% had a bachelor’s degree, and 26.6% had a master’s degree, while 17.1% had a postgraduate degree. A total of 14.7% of the sample were students, 61.3% were workers, and 24.2% were unemployed and retired. A total of 46.8% declared themselves to be religious people, 31.8% to be atheists, and 21.4% to be agnostics. Participants were finally asked to express their “political alignment” in the conflict, i.e., they could choose if they felt more aligned with Ukraine (55.2%), Russia (12.4%), or neither of them (32.4%) (Table 1).

### 2.4. Data Analysis

**Figure 1:**
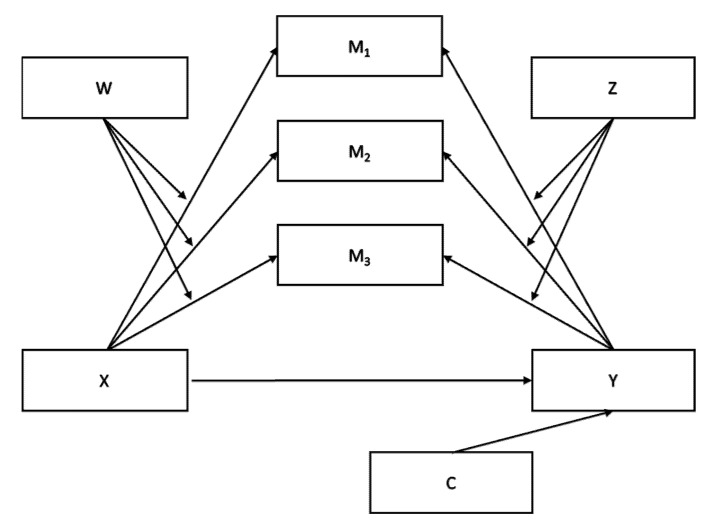
Proposed multiple moderated mediation model (Model 21). Note. X, independent variable; Y, dependent variable; M1, mediator 1; M2, mediator 2; M3, mediator 3; W, moderator 1; Z, moderator 2; C = covariate.

IBM SPSS software version 27 (IBM, Armonk, NY, USA) was used for statistical analysis, and statistical significance was defined as *p* < 0.05. First, we conducted a descriptive analysis of the sample (Table 1). One-way ANOVA was conducted to test for differences in descriptive statistics (i.e., nationality, educational degree, religious belief, marital status, and work) and perceived level of information about the conflict between groups. Then, we tested the internal consistency of the instruments by means of Cronbach’s alphas, with the results showing an internal consistency with an alpha ranging from 0.516 to 0.908. A correlation analysis was performed between the results of the AR to conflict-related events scale with all the other scales, followed by a multiple moderated mediation model analysis (Model 21; Figure 1), which was carried out using Process (version 4.0; Hayes, 2022) to investigate the effect of the independent variables on the dependent variable (political reactions to conflict-related events).

## 3. Results

One-way ANOVA concerning differences between groups in demographic variables showed no statistically significant results for nationality (F_(2)_ = 0.074; *p* = 0.93), educational level (F_(2)_ = 1.203; *p* = 0.30), religious belief (F_(2)_ = 0.505; *p* = 0.60), marital status (F_(2)_ = 0.365; *p* = 0.70), or work (F_(2)_ = 1.51; *p* = 0.22). However, statistically significant differences emerged between groups on perceived level of information about the conflict (F_(2)_ = 5.943; *p* < 0.01). The results showed higher mean scores in both the Russia (M = 3.79, SD = 1.050) and Ukraine (M = 3.64, SD = 0.946) political alignment group compared to neither political alignment group (M = 3.37; SD = 0.977).

Preliminary data analysis did not show nonnormal variables; variable descriptors are summarised in Table 2.

Pearson’s correlation analysis showed that the AR score was significantly and positively correlated with all negative emotions (i.e., shame, disgust, anger), while both ERQ and BEIS variables did not show significant results. For clarity, we report all correlations and statistical results in Table 3. In more detail, AR shows a weak positive correlation with shame, disgust, and anger.

Subsequently, we tested the prediction concerning the link between cognitive reappraisal, negative emotions (i.e., disgust, anger, shame), and AR for levels of appraisal of one’s own emotion and for each group considered (i.e., Russia, Ukraine, and neither political alignment).

The ERQ_CR seems to enhance feelings of negative emotion towards the Russian–Ukrainian war (disgust: *B* = 0.61, *p* < 0.05; anger: *B* = 0.72, *p* < 0.01; shame: *B* = 0.64, *p* < 0.05), providing support for H1. As proposed in H2, BEIS_OwnE significantly negatively moderated the effect of ERQ_CR on negative emotion (disgust: *B* = −0.14, *p* < 0.05; anger: *B* = −0.20, *p* < 0.01; shame: *B* = −0.16, *p* < 0.05), as shown in Table 4. Next, simple slopes for ERQ_CR to negative emotion on different levels of the moderator were examined. Specifically, the results showed that the relationship between ERQ_CR and both disgust and shame was stronger for individuals with a lower BEIS_OwnE score (*B* = 0.19, *p* < 0.05; *B* = 0.17, *p* < 0.05, respectively), while the relationship with anger was stronger for individuals with a higher BEIS_OwnE score (*B* = −0.17, *p* < 0.05) (see Appendix A). The covariate variable considered in the model (i.e., perceived level of information about the conflict) showed no statistically significant results in disgust and shame (*p* = 0.07; *p* = 0.91, respectively), while the anger variable showed a significant effect (*B* = 0.25, *p* < 0.001).

Disgust was negatively related to AR (*B* = −0.09, *p* < 0.01), and shame was positively related to AR (*B* = 0.10, *p* < 0.01), but anger showed no significant results, as shown in Table 4. As advanced in H3, political alignment significantly moderated the effect of negative emotions on AR (disgust: *p* < 0.05; anger: *p* < 0.001; shame: *p* < 0.05). Next, simple slopes for negative emotion to AR on different levels of the moderator were examined. Specifically, the results showed that the relation between both disgust and shame was stronger for the neither political alignment group (*B* = −0.09, *p* < 0.01; *B* = 0.10, *p* < 0.01, respectively), while the relationship with anger was stronger for the Ukraine political alignment group (*B* = 0.14, *p* < 0.001) (see Appendix A). The perceived level of information about the conflict showed no statistically significant results on AR (*p* = 0.87).

**Figure 2:**
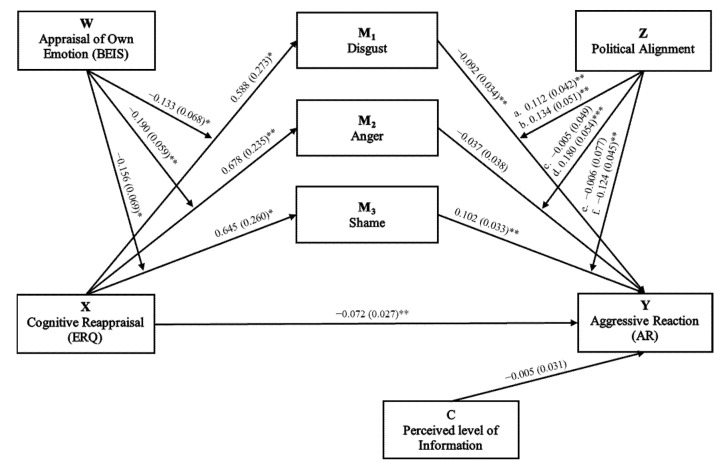
Multiple moderated mediation model in which the mediators were considered in parallel. Numbers represent standardised coefficients. Numbers within parentheses represent standardised errors. Note: X, independent variable; Y, dependent variable; C, covariate; M1, mediator 1; M2, mediator 2; M3, Mediator 3; W, Moderator 1; Z, Moderator 2; a., M1 × Z1; b., M1 × Z2; c., M2 × Z1; d., M2 × Z2; e., M3 × Z1; f., M3 × Z2; * *p* < 0.05; ** *p* < 0.01; *** *p* < 0.001.

Furthermore, as shown in Table 5, a significant direct effect emerged for ERQ_CR on AR (*B* = −0.07, *p* < 0.01). This evidence supports negative emotions as partial mediators of the relationship between ERQ_CR and AR. The model also showed significant indirect effects; in particular, the relationship between ERQ_CR and AR was significantly mediated by disgust and shame in the neither political alignment group at lower BEIS_OwnE scores (*B* = −0.02, 95%CI LL = −0.043, UL = −0.0004; *B* = 0.02, 95%CI LL = 0.0006, UL = 0.043), while anger mediates this relationship in the Ukraine political alignment group at higher BEIS_OwnE scores (*B* = −0.03, 95%CI LL = −0.049, UL = −0.003). The results suggest that individuals with high ERQ_CR experienced negative emotions based on their level of BEIS_OwnE and higher AR based on the political alignment group. The final mediation model (Figure 2) explained 49% of the variance in AR (Table 4).

## 4. Discussion

To test our hypothesis, we used a model that echoes the concepts already expressed in previous studies [7,8,31] regarding the effect of emotional regulation in the Israeli–Palestinian conflict. Specifically, we expected that emotional regulation might affect AR in the Russian–Ukrainian conflict. As our research, unlike Halperin’s [31], did not take place directly in the territory of the conflict, we hypothesised that the AR might be vicariously influenced by the political alignment expressed towards Russia, Ukraine, or neither of them rather than simply by negative emotions. Furthermore, based on the process of emotional regulation [39], we hypothesised that cognitive reappraisal might have an effect not only directly on the AR but also on decreasing the experience of negative emotions through the recognition of one’s own emotions.

### 4.1. Emotion and Emotion Regulation

Our results showed that cognitive reappraisal seems to increase disgust, anger, and shame felt towards the Russian–Ukrainian conflict; this finding can be explained by the goal or motivation for which it is used [49,50,51]. In our study, the emotion is closely related to a war where the sample is not personally involved, and people were asked to refer to a specific affective state, so in the absence of training on cognitive reappraisal, there may not be an underlying desire to minimize the emotion felt [31,52]. This type of result can be explained by the distance from the conflict, as happens, for example, in the case of bombings reported by the mass media that affect social identities [53]. Moreover, war can trigger an ingroup and outgroup categorisation if a threat is perceived to be related to personal safety or towards the country (collective national identity), leading to the support of aggressive policies against the outgroup. However, the power of social identity can differ among individuals, leading to no change in political or social attitudes [53,54,55]. Following an event that acts on social identity, group-based emotions should be elicited differentially, depending on how people categorise themselves [56,57,58,59].

### 4.2. Political Alignment and Emotional Intelligence

Our results confirmed an effect of political alignment on the existing relationships among negative emotions experienced towards conflict and the subsequent AR. This link has been disclosed both by those who take a side (i.e., Ukraine) and by those who do not. However, there is a difference in the emotion felt that leads the individual towards an AR. Those who do not take sides with either nation showed increased AR when the emotion felt is shame, while disgust would seem to “protect” from agreeing to aggressive policies. In contrast, those aligned with Ukraine showed increased AR, as the emotion of anger increased, for instance, the idea that NATO should intervene militarily to end the war. In the NATO context, American and European military forces seem to have the resources to react, triggering emotions of anger rather than fear that would prompt conciliatory policies [59]. However, this reaction relies on the ability to recognise and regulate one’s own emotions; in fact, our results showed how people with higher abilities of emotion recognition seemed to cognitively reassess the situation by decreasing the experience of anger. In contrast, in people with a lower ability to recognise their emotions, an increase in cognitive reappraisal would appear to be associated with higher levels of disgust and shame, moderating the relationship between emotional regulation and changes in the experience of emotions. Difficulties in emotional understanding and verbalising may lead to the persistence of unpleasant feelings even after cognitive reappraisal [38,43,44,60]. Reflecting and trying to change the meaning of the conflict could lead to an increase in its awareness by enhancing the experience of disgust and shame that could be directed at the general situation. Instead, anger may be a more connected emotion to others, and its reappraisal could lead to modification towards other forms of emotion, such as disgust or shame [21].

## 5. Conclusions

In this paper, we wanted to explore the role of adaptive emotion regulation strategies in individual disposition to aggressive behaviours toward the outgroup for the current Russian–Ukrainian war resolution. Moreover, we hypothesized that emotional intelligence and political alignment might have a role to explain the relationship among emotion regulation and aggressive policies in the context of conflicts besides the specific emotion involved in the model already used in the literature. We emphasized the importance of recognising one’s own emotions in intergroup conflicts, hypothesising that siding with one nation rather than another can generate different emotions and, therefore, different behaviours.

Just as shown in other warfare contexts, our results showed that emotions elicited by conflict (i.e., disgust, anger, shame) can help explain how the use of emotional regulation strategies (i.e., cognitive reappraisal) may affect the propensity toward aggressive policies. Previous studies showed that adaptive emotional regulation strategies decrease the experience of negative emotions; however, in the present study this seems to be true for some emotions (anger) while not for others (shame and disgust), according to emotion recognition ability. Moreover, the effect of specific emotions in increasing (i.e., anger and shame) or decreasing (disgust) support for aggressive policies changes according to political alignment with a specific party involved in the conflict.

These results are useful to intervene to support those people who, although not directly involved in the conflict, experience emotions that, if not elaborated on, can become invalidating. Moreover, although it may be useful to develop training programs for increasing cognitive reappraisal, to decrease aggression towards the outgroup and promote a tendency towards conciliatory policies, this study provides an insight into the development of programs that consider all the capacities needed to manage the individual’s emotional experience.

The research also provided useful results for future studies that might explore the effect of the media on emotional regulation in conflicts that are distant from the target population as well as the effect of political orientation on the perception of conflict rather than on aggressive politics.

Despite this, the research has some limitations. Replicating a study in a different context from the original research has theoretical effects: the conflict experienced on location compared to that reported by the media has a different effect on the emotions experienced and their regulation, particularly about the perception of “victims” and “perpetrators”. In addition, political orientation, which has been present in previous research, and media exposure were not investigated. In the first case, the choice not to include it was made because the sample is not directly involved in the conflict, thus avoiding ideology bias. Regarding the media, it is relevant not only what news is reported but also how much news is received and looked for influences the emotions experienced; particularly, in emergency situations, a media-bombing situation occurs, in which all news is focused only on the topic of the emergency, with important effects on the experience and regulation of emotions. Finally, the sample that reported supporting Russian policies is much smaller than the other two groups, making a methodologically meaningful comparison between the groups difficult.

## Figures and Tables

**Table 1:** Demographic characteristics of the sample.

Age Range	18–89 Years	M = 40.03	SD = 13.83
		*n*	%
Gender	Female	330	64.8%
	Male	175	34.4%
	Other	4	0.8%
Educational Degree	Postgraduate degree	87	17.1%
	Master’s degree	135	26.6%
	Bachelor’s degree	92	18.0%
	High School	174	34.2%
	Secondary School	21	4.1%
Religious Belief	Agnostic	109	21.4%
	Atheist	162	31.8%
	Religious	238	46.8%
Marital Status	Married	162	31.8%
	Separate	21	4.1%
	Divorced	7	1.4%
	Single	103	20.2%
	Widow	7	1.4%
	Non-cohabiting partner	88	17.3%
	Cohabiting partner	121	23.8%
Work	Worker	311	61.3%
	Student	75	14.7%
	Unemployed/retired	123	24.2%
Political Alignment	Russia	63	12.4%
	Neither	165	32.4%
	Ukraine	281	55.2%

**Table 2:** Descriptive statistics for the aggressive reactions (AR), negative emotions, emotion regulation questionnaire (ERQ), and brief emotional intelligence scale (BEIS)-10.

	M (SD) *n* = 509	Median *n* = 509
AR	2.28 (0.85)	2.25
Disgust	3.49 (1.39)	4.00
Anger	3.04 (1.28)	3.00
Shame	2.33 (1.46)	2.00
ERQ_CR	4.77 (1.02)	4.83
ERQ_ES	3.46 (1.23)	3.50
BEIS	3.78 (0.47)	3.80
BEIS_OwnE	3.78 (0.73)	4.00
BEIS_OthE	3.85 (0.70)	4.00
BEIS_ROwn	3.69 (0.64)	3.50
BEIS_ROth	3.52 (0.84)	3.50
BEIS_EU	4.08 (0.67)	4.00
Info	3.57 (0.98)	3.00

Note. AR, Aggressive Reaction to Conflicted-Related Events Scale; ERQ_CR, Cognitive Reappraisal; ERQ_ES, Expressive Suppression; BEIS, Brief Emotional Intelligence Scale; BEIS_OwnE, Appraisal of One’s Own Emotions Subscale; BEIS_OthE, Appraisal of Others’ Emotions Subscale; BEIS_ROwn, Regulation of One’s Own Emotions Subscale; BEIS_Roth, Regulation of Other’s Emotions Subscale; BEIS_EU, Utilisation of Emotion Subscale; Info, perceived level of information about the conflict.

**Table 3:** Correlation coefficients (Pearson’s r) between the AR and negative emotions, ERQ, and BEIS (*n* = 509) scores.

	Disgust	Anger	Shame	ERQ_CR	ERQ_ES	BEIS	BEIS_OwnE	BEIS_OthE	BEIS_ROwn	BEIS_ROth	BEIS_EU	Info
AR	0.240 **	0.281 **	0.206 **	−0.050	0.053	0.014	−0.047	0.051	0.050	0.028	−0.038	0.058

Note. AR, Aggressive Reaction to Conflicted-Related Events Scale; ERQ_CR, Cognitive Reappraisal; ERQ_ES, Expressive Suppression; BEIS, Brief Emotional Intelligence Scale; BEIS_OwnE, Appraisal of One’s Own Emotions Subscale; BEIS_OthE, Appraisal of Others’ Emotions Subscale; BEIS_ROwn, Regulation of One’s Own Emotions Subscale; BEIS_ROth, Regulation of Other’s Emotions Subscale; BEIS_EU, Utilisation of Emotion Subscale; Info, perceived level of information about the conflict; ** = *p* < 0.01.

**Table 4:** Model coefficients for the multiple moderated mediation analysis (*n* = 509).

Predictor	Disgust		Anger		Shame		AR	
	*β* (*SE* HC0)	*p*	*β* (*SE* HC0)	*p*	*β* (*SE* HC0)	*p*	*β* (*SE* HC0)	*p*
Constant	0.058 (1.305)	0.96	−1.542 (1.101)	0.16	−0.388 (1.186)	0.74	2.374 (0.216)	<0.001
ERQ_CR (X)	0.606 (0.275)	<0.05	0.716 (0.238)	<0.01	0.644 (0.261)	<0.05	−0.072 (0.027)	<0.01
BEIS_OwnE (W)	0.682 (0.326)	<0.05	1.011 (0.271)	<0.001	0.660 (0.311)	<0.05		
X × W	−0.137 (0.068)	<0.05	−0.196 (0.060)	<0.01	−0.156 (0.069)	<0.05		
Disgust (M_1_)							−0.092 (0.034)	<0.01
Anger (M_2_)							−0.037 (0.038)	0.32
Shame (M_3_)							0.102 (0.033)	<0.01
Z_1_							−0.841 (0.192)	<0.001
Z_2_							0.163 (0.199)	0.41
M_1_ × Z_1_							0.112 (0.041)	<0.01
M_1_ × Z_2_							0.134 (0.051)	<0.01
M_2_ × Z_1_							−0.004 (0.050)	0.93
M_2_ × Z_2_							0.181 (0.054)	<0.001
M_3_ × Z_1_							−0.005 (0.078)	0.95
M_3_ × Z_2_							−0.124 (0.045)	<0.01
Info	0.121 (0.066)	0.07	0.249 (0.060)	<0.001	−0.007 (0.066)	0.91	−0.005 (0.031)	0.87
R^2^	0.019 *		0.062 ***		0.011			0.49 ***
*F* HC0 (df)	2.448 (4.000)		8.585 (4.000)		2.140 (4.000)		74.637 (13.000)	
ΔR^2^	0.008 *		0.019 **		0.009 *		M_1_ × Z	0.007 *
							M_2_ × Z	0.013 ***
							M_3_ × Z	0.008 *

Note. AR, aggressive reaction; ERQ_CR, cognitive reappraisal; BEIS_OwnE, Appraisal of One’s Own Emotions Subscale; Z, political alignment group; Z_1_ and Z_2_, dummy variables in two-way interaction. Z_1_, comparing neither and Russia political alignment on AR; Z_2_, comparing neither and Ukraine political alignment on AR; Info, perceived level of information about the conflict. Bootstrap sample size = 5000 (two-tailed); * = *p* < 0.05; ** = *p* < 0.01; *** = *p* < 0.001.

**Table 5:** Bootstrap direct and conditional indirect effect of reappraisal strategies on AR at values of appraisal of one’s own emotion and political alignment (Model 21).

Direct Effect			*β* (*SE* HC0) −0.072 (0.027)		95% Boot CI (LL; UL) (−0.124; −0.020)			
Indirect Effect	Levels of Appraisal of One’s Own Emotion	Political Alignment	Disgust		Anger		Shame	
			*β* (Boot*SE*)	95% Boot CI (LL; UL)	*β* (Boot*SE*)	95% Boot CI (LL; UL)	*β* (Boot*SE*)	95% Boot CI (LL; UL)
	Low	Neither	−0.017 (0.012)	(−0.043; −0.0004)	−0.004 (0.006)	(−0.019; 0.006)	0.017 (0.011)	(0.0006; 0.043)
	Low	Russia	0.004 (0.006)	(−0.006; 0.017)	−0.005 (0.006)	(−0.020; 0.004)	0.016 (0.016)	(−0.008; 0.053)
	Low	Ukraine	0.008 (0.009)	(−0.007; 0.027)	0.017 (0.012)	(−0.007; 0.043)	−0.004 (0.006)	(−0.018; 0.008)
	Medium	Neither	−0.008 (0.007)	(−0.025; 0.003)	0.001 (0.003)	(−0.005; 0.009)	0.006 (0.008)	(−0.008; 0.024)
	Medium	Russia	0.002 (0.003)	(−0.003; 0.010)	0.001 (0.003)	(−0.005; 0.009)	0.005 (0.009)	(−0.008; 0.027)
	Medium	Ukraine	0.004 (0.005)	(−0.004; 0.016)	−0.004 (0.009)	(−0.022; 0.013)	−0.001 (0.003)	(−0.009; 0.004)
	High	Neither	0.001 (0.008)	(−0.014; 0.017)	0.006 (0.008)	(−0.007; 0.024)	−0.006 (0.010)	(−0.025; 0.014)
	High	Russia	−0.0002 (0.003)	(−0.005; 0.006)	0.007 (0.007)	(−0.004; 0.024)	−0.006 (0.011)	(−0.031; 0.014)
	High	Ukraine	−0.0004 (0.004)	(−0.010; 0.009)	−0.025 (0.012)	(−0.049; −0.003)	0.001 (0.004)	(−0.005; 0.011)

**Figure A1:**
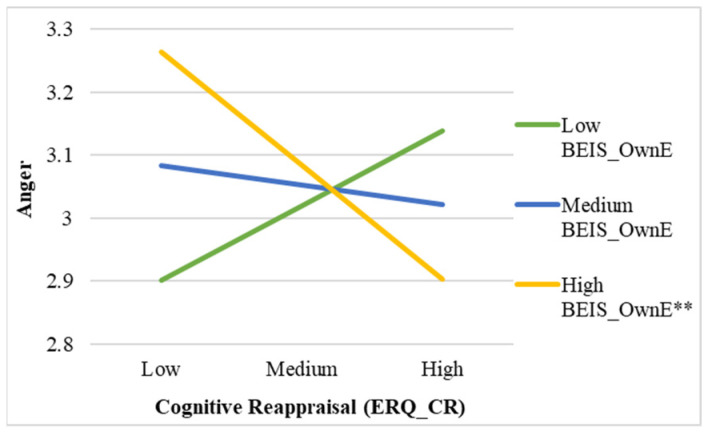
Plot of the relationship between Cognitive Reappraisal and Anger at low (−1 SD), medium (mean), and high (+1 SD) Appraisal of Own Emotion score; ** = *p* < 0.01.

**Figure A2:**
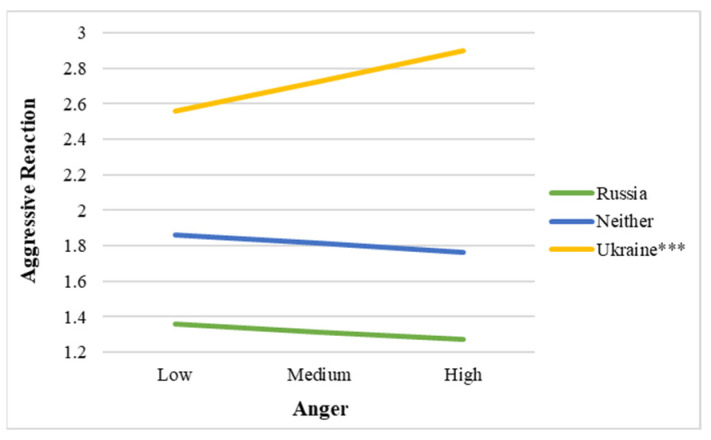
Plot of the relationship between Anger and Aggressive Reaction on Russia, Ukraine, and Neither Political Alignment; *** = *p* < 0.001.

**Figure A3:**
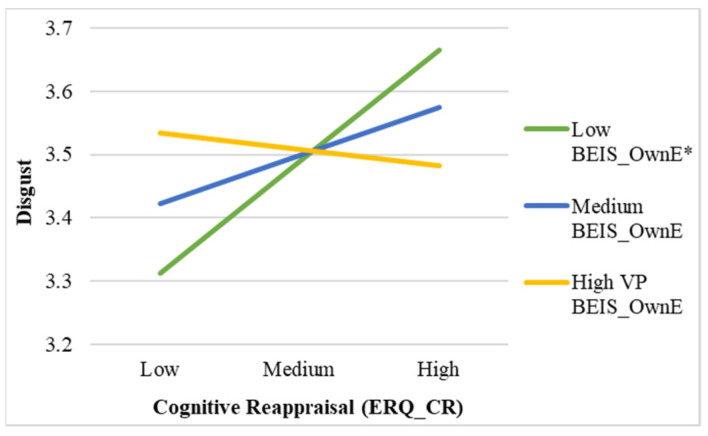
Plot of the relationship between Cognitive Reappraisal and Disgust at low (−1 SD), medium (mean), and high (+1 SD) Appraisal of Own Emotion score; * = *p* < 0.05.

**Figure A4:**
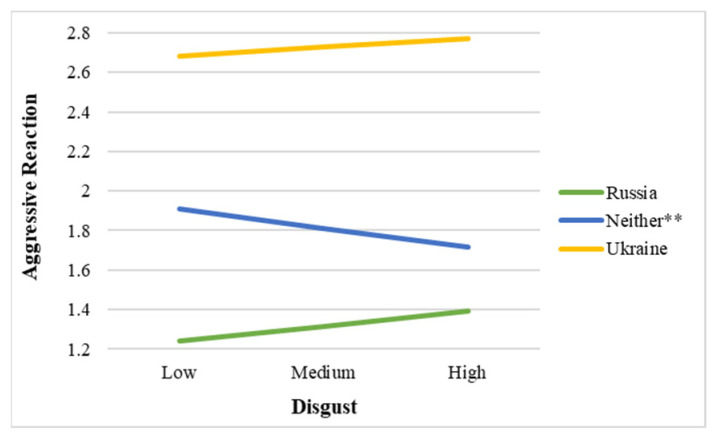
Plot of the relationship between Disgust and Aggressive Reaction on Russia, Ukraine, and Neither Political Alignment; ** = *p* < 0.01.

**Figure A5:**
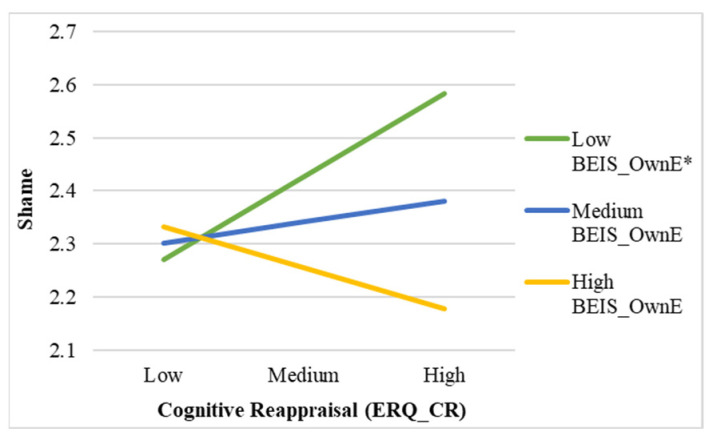
Plot of the relationship between Cognitive Reappraisal and Shame at low (−1 SD), medium (mean), and high (+1 SD) Appraisal of Own Emotion score; * = *p* < 0.05.

**Figure A6:**
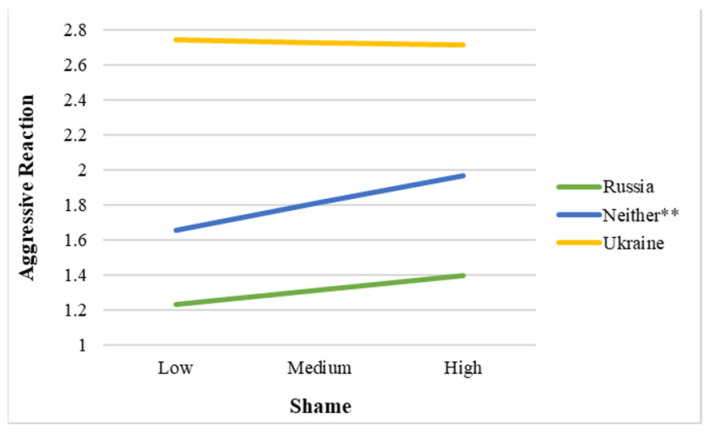
Plot of the relationship between Shame and Aggressive Reaction on Russia, Ukraine, and Neither Political Alignment; ** = *p* < 0.01.

## Data Availability

Data are available on request due to privacy restrictions.
